# Prevalence of Genotype Patterns Associated With High-Risk Human Papillomavirus in Cervical Lesions

**DOI:** 10.7759/cureus.58300

**Published:** 2024-04-15

**Authors:** Varun Kothari, Shivani Khullar, Hemavaishnave TS

**Affiliations:** 1 Microbiology, Dr. Sampurnanand Medical College, Jodhpur, IND; 2 Internal Medicine, Aarya Polyclinic, Mumbai, IND

**Keywords:** real-time pcr, high-risk hpv, viral transport medium, cervical lesions, human papillomavirus genotypes

## Abstract

Background

Cervical lesions, often linked to high-risk human papillomavirus (HR-HPV) infection, pose a significant public health concern globally. Human HPV is considered the principal etiological factor in the development and transformation of pre-malignant lesions of the cervix leading to cervical carcinoma. The global distribution of HPV genotypes exhibits significant variability, liable to be influenced by the intricate interplay of geographical and biological factors among different HPV types and host immunogenetic elements. Owing to limited research addressing the genotypic distribution of HR-HPV in females with cervical lesions in the western zone of India, this study aims to bridge this gap by providing the prevalence of HR-HPV genotypes in women diagnosed with cervical lesions in this zone.

Methodology

This observational, cross-sectional study was performed in the Laboratory for Genotype Detection in the western zone of India. The study population comprised a total of 215 females in the age range of 18 to 60 years. A thorough examination of clinical specimens was conducted employing molecular techniques for HPV genotyping using TRUPCR® HPV-HR with a 16/18 Genotyping Kit. DNA Extraction was done using 3B Blackbio Biotech India Ltd. as per the standard protocol. The statistical analysis was performed using SPSS version 28 (IBM Corp., Armonk, NY, USA), and continuous variables were compared using Student’s t-test.

Results

The overall prevalence of HR-HPV in women with cervical lesions in the western zone of India was 62.32% (134/215). The most prevalent genotype was HPV16 at 92/134 (68.65%), followed by HPV18 at 33/134 (24.62%) and HPV52 at 9/134 (6.7%). The overall prevalence of single type was 56.71% (76/134). The most prevalent genotype combination after HPV18 + 59 (29.85%) at 40/134 was HPV52 + 39 + 51 (13.43%) at 18/134. HR-HPV infection was found to be significantly (p < 0.05) associated with factors such as having three or more children, having a lower socioeconomic status, residing in rural areas, and being in a pre-menopausal stage.

Conclusions

This study focused on assessing the prevalence of the genotypes associated with HR-HPV infection, providing valuable insights into the epidemiology of cervical lesions in the western zone of India. The findings revealed high-risk genotype HPV16 to be the most prevalent type, followed by HPV18 and HPV52. The most prevalent genotype combinations were HPV18 + 59 and HPV52 + 39 + 51. The results of the study would potentiate the wealth of epidemiological data related to HPV infection in cervical lesions and could be employed for guiding future interventions and preventive strategies through orientation programs.

## Introduction

Human papillomavirus (HPV) is the predominant pathogen and one of the most common causes of anogenital cancers worldwide at present. Cervical lesions associated with high-risk human papillomavirus (HR-HPV) infection represent a substantial global public health concern due to their role in the development of cervical cancer as identified in over 90% of cervical cancer cases [1]. According to the Global Cancer Statistics (GLOBOCAN) 2020 statistics, worldwide, a total of 604,127 new cases of cervical cancer and 341,831 deaths were reported in 2020, making it the fourth most common cancer in females. There is a high burden of cervical cancer in developing countries, comprising 83% of all new cases and 85% of cervical cancer deaths [2].

The link between persistent HR-HPV infections and the progression of cervical lesions to cervical cancer is well-established in the scientific literature, and the incidence of invasive cervical cancer continues to be high, particularly in western India [3,4]. HPV, especially the high-risk genotype, has been identified as a leading cause of cervical intraepithelial neoplasia and cervical carcinoma, emphasizing the critical importance of understanding the epidemiology and genotype patterns of HR-HPV in affected populations [5].

Anogenital infections are associated with around 40 genotypes of HPV [6]. These strains are categorized into the following three groups based on their oncogenic potency: high-risk, low-risk, and intermediate-risk types [6]. High-risk types include HPV16, 18, 31, 33, 35, 39, 45, 51, 52, 56, 58, 59, 68, 73, and 82, as they are predominantly found in high-grade squamous intraepithelial lesions. Low-types, including HPV6, 11, 40, 42, 43, 44, 54, 61, 70, 72, and 81, cause benign warts. Intermediate-risk types include HPV23, 53, and 66 [7].

Persistent HR-HPV infection is a primary cause of cervical lesions [8,9]. As the western zone of the country is home to Asia’s largest slum, poor hygiene, demographics, socioeconomic and structural barriers, and inadequate medical facilities are responsible for ineffective, organized, and opportunistic cervical cytology screening. Owing to all these, HPV infection majorly remains undetected. Hence, the incidence of cervical lesions in this zone is proportionately escalated to an estimated annual percent change of 1.8% (95% confidence interval (CI) = −2.0, −1.6), as per the Mumbai registry [10]. Further, there is a lack of education, widespread screening initiatives, public awareness campaigns, orientation programs, and vaccination drives. The prevalence of child marriages amounts to early age at marriage, early sexual activity, or early coitarche which become factors for the persistence and progression of HPV infection toward cervical cancer [9]. This zone of India exhibits a distinct cultural, climatic, and geographical landscape compared to other parts of the country. Therefore, it becomes crucial to ascertain the genotype distribution for better implementation and improved execution of routine cervical screening programs.

The epidemiological spread of HPV infection and its associated genotypes, as documented in various studies [10-14], exhibit notable variations, influenced by diverse factors such as geographic location, socioeconomic status, cultural practices, and genetic variability of the viral genome. Additionally, intrinsic individual factors such as age, gender, anatomical site, and health condition contribute to the morbidity and mortality associated with HPV infections [15-17].

Reports from studies [3-6,11-14] conducted in various regions of India indicate significant differences in the prevalence of HPV infection and the pattern of genotype distribution. HR-HPV prevalence in patients with cervical cancer was 87.8% in Andhra Pradesh with the pattern of genotypes reported as HPV16 (66.7%), HPV18 (19.4%), HPV33 (5.6%), HPV35 (5.6%), HPV45 (5.6%), HPV52 (2.8%), HPV58 (2.8%), HPV59 (2.8%), and HPV73 (2.8%) [11]. With 99.4% HR-HPV prevalence in rural Tamil Nadu, the prevailing types identified were HPV16 and 56, with HPV31, 33, and 18 following in succession [6]. As reported by Senapati et al. in eastern India [8], HPV16 prevalence was 87.28%, followed by HPV18 (24.56%) and HPV51 (3.46%). The overall prevalence of single type was 76.58%, and the most frequent genotype combination after HPV16 + 18 (9.4%) was HPV16 + 66 + 68 (2.7%). HPV16 positivity in 56% and HPV18 in 15% of cases were reported from Gujarat, whereas 6% of the cases showed co-infection with both [3]. HPV16 was detected in 81.7% of cervical cancer cases in rural central India [11]. Similarly, in 36.84% of HPV-positive females in West Bengal [12], the prevalence of HPV16 with 18 was 79.37% compared to other HPV types (20.63%). A prevalence of HPV16 (77.08%) and HPV18 (16.67%) was reported by Kumar et al. [13] in Bihar. These variations are linked to the diverse socioeconomic and geoclimatic conditions in specified areas. Consequently, there is a need for a comparable assessment of HPV genotypic distribution in various geographical zones across the country.

In the western zone, despite the high burden of cervical cancer, there exists limited available data [3,4] focused on investigating the distribution and genotype patterns of HR-HPV among females with cervical lesions hindering the development of targeted prevention and intervention strategies tailored to the specific needs. The unique sociocultural and demographic characteristics of this geographic region may contribute to distinct patterns of HPV infection, necessitating a zone-specific investigation.

This study aims to provide the prevalence of HR-HPV genotypes among females with cervical lesions in the western zone of India. Through this documentation of prevalent strains, this study seeks to offer essential insights that can help screening programs target specific HR-HPV genotypes, allowing for the tailoring of preventive strategies.

## Materials and methods

Study design and setting

This was an observational, cross-sectional study. The Strengthening the Reporting of Observational Studies in Epidemiology (STROBE) guidelines were used for reporting and preparing the manuscript. The study was conducted at the Laboratory for Genotype Detection from April 2023 to January 2024. The study was approved by the Institutional Ethics Committee for Research on Human Subjects (approval number: RES/IEC/59/2023).

Study population

A total of 215 patients attending the healthcare facilities in the western zone of India were enrolled based on the selection criteria. The sample size was estimated using the standard statistical formula [18] for prevalence study, where n was the sample size, Z was the statistic corresponding to the level of confidence, P was the expected prevalence as per Gupta et al. [4], and d was the precision (0.10 corresponding to the effect size) assuming a confidence level of 95%.

Selection criteria

Females aged 18 to 60 years experiencing abnormal discharge per vagina, post-coital hemorrhage, irregular menstruation, dyspareunia, dysuria, and pruritus were included in the study. Pregnant women, those with active vaginal bleeding, and those using intrauterine devices were excluded. Written informed consent was obtained from each participant.

Data sources and measurement of variables

The demographic and clinical data from all patients were recorded in the case record form (proforma), wherein details about the age at conception, marital and menopausal status, parous state, residential location, socioeconomic status, HPV infection history, or any other medical conditions such as human immunodeficiency virus infection was documented.

**Figure 1:**
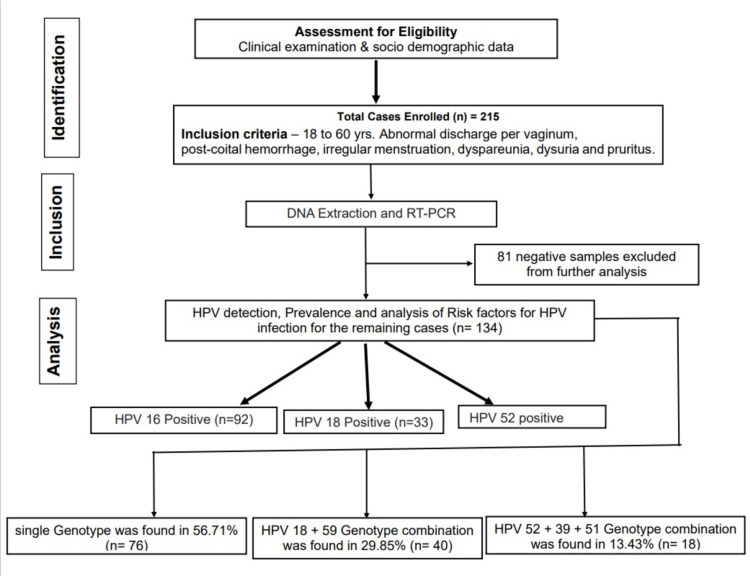
STROBE flow diagram . HPV = human papillomavirus; RT-PCR: real-time polymerase chain reaction

The enrollment of cases is represented in a STROBE flow diagram in Figure 1. The study sample was adequate within the study duration and the potential for confounding was ensured considering that the data was obtained from representative settings in which healthcare is practiced in this zone.

Sample Collection

Clinical samples were collected from females undergoing cervical screenings in healthcare facilities in western India. Following aseptic precautions, right at the commencement of the examination, a cyto-brush was inserted into the external cervical os and gently rotated to scrape cells from both the ectocervix and endocervix. The brush head was then removed and placed into a viral transport medium vial with preservative fluid, which was subsequently transported to the Laboratory for Genotype Detection.

DNA Extraction and HPV Genotype Detection

Genomic DNA was extracted from the collected samples using a DNA extraction kit by 3B Blackbio Biotech India Ltd. according to the standard protocol [19]. The amplification of the HPV genome was performed by real-time polymerase chain reaction (RT-PCR) technique employing the TRUPCR® HPV HR with 16/18 Genotyping Kit for the detection and genotyping of HPV DNA in clinical specimens. In this kit, fluorescent reporter dye probes specific for the detection of high-risk HPV14 genotypes (16, 18, 31, 33, 35, 39, 45, 51, 52, 56, 58, 59, 66, and 68) with a focus on genotypes 16 and 18 are utilized. Within this kit, three separate reactions are conducted simultaneously in three tubes. The first reaction targets HR-HPV genotypes 16, 31, 33, 35, 52, 58, 51, 56, and 66, utilizing the fluorescein amidite (FAM) channel for detection. In the second reaction, HR-HPV genotypes 18, 45, and 59 are detected via the FAM channel, with additional genotyping of HPV18 using the hexa-chloro-fluorescein dye and an endogenous internal control in the TEXAS RED channel, facilitating the exclusion of unreliable results. Finally, the third reaction identifies either HR-HPV genotype 39 or 68 using the FAM channel, accompanied by the genotyping of HPV16 in the TEXAS RED channel.

Data analysis

The recorded data were abstracted into Microsoft Excel and statistically analyzed using SPSS Statistics for Windows version 28.0 (IBM Corp., Armonk, NY, USA). Continuous variables were expressed as mean ± standard deviation and were compared using Student’s t-test. The standard contingency table method was employed to determine the association between demographic variables, clinical features, and the occurrence of HPV infection in various samples using the chi-square test. A two-sided p-value was computed, and the significance threshold was at p < 0.05.

## Results

Demographic data

A total of 215 patients from the western zone of India were included in this study. The mean age was 38.42 years, with a standard deviation of 10.39 years. The age and parity-wise distribution and demographic statistics of the enrolled patients are depicted in Table 1.

**Table 1:** Sociodemographic statistics of patients.

Characteristics	Total (n = 215)	
Age (years) (mean ± SD)	38.42 ± 10.39	
Demographic variable	Number (n)	Percent (%)
Place of residence		
Rural	158	73.48%
Urban	57	26.52%
Education		
Illiterate	55	24.35%
Primary	60	30.00%
Secondary	53	26.60%
Higher education	16	19.05%
Socioeconomic status		
Low	58	26.97%
Middle	115	53.48%
High	42	19.53%
Menstrual status		
Pre-menopausal	134	62.32%
Post-menopausal	81	37.68%
Marital status		
Unmarried	31	14.42%
Married	184	85.58%
Parity		
Nil	12	5.58%
1–2	65	30.23%
More than equal to 3	138	64.18%

HPV infection had a significant association with several factors, including parity of three or more (odds ratio (OR) = 1.85, p = 0.008), poor socioeconomic condition (OR = 1.59, p < 0.0001), rural residence (OR = 21.42, p = 0.0043), and being in a pre-menopausal stage (OR = 1.78, p = 0.0002). Of the 215 females, 184 were married and 31 were unmarried. The majority of cases belonged to rural stratum (158/215, 73.48%) and remaining to urban (57/215, 26.52%). Approximately 54.35% were either illiterate or had only primary education. Regarding menstrual status, out of the 215 cases, 143 (66.51%) were premenopausal and 72 (33.48%) were postmenopausal. Further, 64.18% of cases had a parity of three or more.

Prevalence of HPV genotypes

The prevalence of HPV genotypes among HPV-positive patients is presented in Table 2. A total of 134 cases out of 215 (62.32%) tested positive for HPV while 81 (37.68%) tested negative for HPV.

**Table 2:** Prevalence of HPV genotypes among HPV-positive patients. HPV = human papillomavirus

HPV		HPV16		HPV18		HPV52	
Number of patients (n)	Percent (%)	Number of patients (n)	Percent (%)	Number of patients (n)	Percent (%)	Number of patients (n)	Percent (%)
134	62.32%	92	68.65%	33	24.62%	9	6.7%

The overall prevalence of HPV16 was 68.65% as 92 cases were positive for HPV16. A notable variation existed in the HPV16 prevalence among the patients. HPV16 was the predominant type significantly associated with the severity of the lesion (p = 0.04). As the F-value is the ratio of between-group variation and within-group variation, a large F-value of 31.23 means that between HPV genotype groups, the variation was larger than within-group variation, which can be interpreted as a statistically significant difference (p = 0.036) in the proportion of HPV16 positivity among the patients.

On the other hand, the prevalence of HPV18 was 24.62% as 33 out of 134 cases tested positive for HPV18, and for HPV52, it was 6.7% as 9 of 134 cases were positive for HPV52.

The prevalence of HPV genotype combinations among HPV-positive patients is presented in Table 3. The overall prevalence of the single type was 56.71% (76/134). The study identified the most prevalent genotype combination after HPV18 + 59 with 29.85% (40/134) cases to be HPV52 + 39 + 51 with 13.43% (18/134).

**Table 3:** Prevalence of HPV genotype combinations among HPV-positive patients. HPV = human papillomavirus

HPV single genotype		HPV genotype 18 + 59		HPV genotype 52 + 39 + 51	
Number of samples (n)	Percent (%)	Number of samples (n)	Percent (%)	Number of samples (n)	Percent (%)
76/134	56.71%	40/134	29.85%	18/134	13.43%

## Discussion

In India, cervical cancer ranks as the second most common malignancy in women aged 15 to 44 years, accounting for 14% of all female cancer cases and one-quarter of the global burden [2]. The total number of cervical cancer cases in 2020 in India was 123,907, accounting for 18.3% of all cancer cases among women, while deaths were 77,348, accounting for 18.7% of female cancer deaths [20]. Despite being preventable, cervical carcinoma has emerged as a devastating disease, making it imperative to understand the epidemiology of cervical cancer in India along with the prevalence of HPV genotypes which are crucial parameters to aim for its prevention. The HPV prevalence varies greatly across India among patients with cervical lesions [6,11,15] and is influenced by the geographical location, genetic variability of the viral genome, and sociodemographic factors affecting different HPV genotypes [8-10,17].

This study identified the prevalence of HR-HPV genotypes in women with cervical lesions in the western zone of India employing precise HPV typing of clinical samples. The methodology involved the utilization of sensitive molecular techniques, such as RT-PCR and DNA sequencing, to accurately detect and categorize HR-HPV genotypes. By implementing these diagnostic tools, the study sought to identify the presence of HR-HPV infection and differentiate between various genotypes, enabling a better understanding of the viral landscape within the specific geographic and demographic context.

Pan-India, HPV occurrence of type 16 alone is reported to be 70-90% and HPV18 prevalence generally varies from 3% to 20% [3-6,11-13]. In this study, while categorizing and quantifying the prevalent viral genotypes of HR-HPV, HPV16 emerged as the most prevalent type (68.65%) whereas the prevalence of HPV18 was 24.62%. A similar study reported HPV prevalence statistics in agreement with our observations that HPV16 constituted the majority (79.37%) of HPV infections followed by HPV18, 33, 31, and 45 [12]. HPV prevalence data aligning with our findings was observed in a study performed by Kumar et al., who reported a prevalence of HPV16 (77.08%) and HPV18 (16.67%) [13]. Additionally, consistent with our findings, the prevalence of 66.7% for HPV16 and 19.4% for HPV18 was reported from Andhra Pradesh [11]. Our results contrast with a study conducted in rural Tamil Nadu, where the prevailing types were HPV16 and 56, followed by HPV31, 33, and HPV18 [6].

The higher occurrence of HPV16 compared to other genotypes might be linked to the intricate interplay of biological factors between the virus and host immunogenetics. HR-HPV viral load, notably for HPV-16, can be a molecular biomarker of risk for developing cervical (pre-) cancerous lesions [4]. The primary motive of the present HPV vaccines consisting of viral-like particles of HPV16 is to guard against severe pre-neoplastic cervical lesions which were found to be predominantly and closely associated with the presence of the HPV16 genotype among patients included in this study.

Diverse factors such as residence in rural areas (p < 0.05), poor socioeconomic condition (p < 0.0001), and females in the pre-menopausal stage (p < 0.05) had more propensity for cervical lesions. These findings corroborate well with the study by Srivastava et al., who also showed that the risk of HPV infection was higher in females aged 25 to 34 years, in those married before 20 years of age, and in females with parity ≥4 [5]. It was observed that a high parous state was significantly associated with HPV infection. This may be due to early age at marriages resulting in early childbearing. It has been reported that high parity leads to four times increased risk of cervical carcinoma [5,21].

Further, it has been documented that individuals staying in a rural area have a 20-fold higher risk of acquiring HPV infection [15]. Studies conducted in rural areas of Andhra Pradesh and Sevagram in central India showed 87% and 93% HPV positivity [6,11]. As rural residence is indicative of poor socioeconomic conditions, it reflects limited access to adequate healthcare, potentially contributing to the spread and persistence of HPV infections and consequently raising the risk of developing cancer. These observations suggest the urgent need for screening programs and HPV vaccination in women with low socioeconomic status and those residing in rural areas. The identified factors responsible for the uptake of screening could be a guiding force in deciding how and where tailored interventions may be best targeted.

Hence, the knowledge regarding the prevalence of HR-HPV genotypes makes healthcare practitioners aware of the potential severity and risks associated with specific HR-HPV strains, guiding clinical management and follow-up protocols. This would help in healthcare planning and the development of targeted interventions to address the specific needs of the population in the western zone of India. It can have implications for devising optimal vaccine development and the effectiveness of implementing cancer prevention initiatives. The effectiveness of vaccines lies in the fact that they target and induce immunity against HR-HPVs responsible for cancer.

The strength of the study lies in contributing to the knowledge base on HPV epidemiology by highlighting the prevalent strains. The data on high-risk genotypes identified in our study would benefit the public health initiatives aimed at reducing the burden of HPV-related cervical lesions by allowing for the tailoring of preventive strategies, awareness programs, and educational campaigns to target the specific HR-HPV genotypes predominant in the western zone of India. The study also offers a foundation for future research and post-vaccination surveillance.

Limitations of the study

As it is a cross-sectional study, it only provides information on HPV genotypes without accounting for the distribution of other viral genotypes. Another bottleneck in this study was the exclusion of pregnant women which was a constraint during sampling.

## Conclusions

This study revealed a high prevalence of HR-HPV genotypes in cervical lesions with HPV16 as the most prevalent single type in the western zone of India, followed by HPV18 and HPV52. In mixed HPV genotype infections, the prevalence of HPV18 + 59 was higher than other combinations. This information based on zonal HPV epidemiological data would contribute to the assessment of the risk of cervical cancer among females and to targeted public health interventions such as effective screening modalities and vaccination strategies to reduce the cervical cancer burden in India.
